# A scoping review on active vs. passive range of motion approaches to treat heterotopic ossification at the elbow

**DOI:** 10.3389/fresc.2024.1327417

**Published:** 2024-06-05

**Authors:** Patricia Siegel, Shanna Smith, Emily Stark, Cole Burns, Timothy P. Dionne

**Affiliations:** Occupational Therapy Graduate Program, Department of Pediatrics, School of Medicine, University of New Mexico, Albuquerque, NM, United States

**Keywords:** heterotopic ossification, occupational therapy, physical therapy, elbow contracture, joint contracture

## Abstract

**Objective:**

The objective of this scoping review is to synthesize and clarify literature on the effectiveness of active and passive range of motion therapy techniques to address range of motion in people with heterotopic ossification (HO), and to provide guidance to therapists in clinical decision-making based on current evidence.

**Method:**

To find articles that included therapeutic interventions to maintain or improve range of motion in people with heterotopic ossification, the authors searched the following databases: Cochrane Database of Systematic Reviews, PubMed, CINAHL, PsychINFO, Web of Science, and OTSeeker. To ensure that the search was comprehensive, the authors also searched Burns and Trauma, Burns Journal, Burns Open, and the Journal of Hand Therapy. Searches were limited to peer-reviewed articles published in the English language. No publication date limits were set. The Physiotherapy Evidence Database PEDro scale was utilized to measure the validity of the methodological quality of each article.

**Results:**

Five studies met the inclusion criteria.. Two studies emphasized that passive range of motion was effective in less than 50% of their subjects, while the other three studies utilized active range of motion only, reporting 50% of patients did not require surgery.

**Discussion/conclusion:**

There is insufficient evidence to determine effective therapeutic management of HO and the literature that does exist is contradictory and inconclusive. Future research is necessary to determine if any effectiveness of manual therapeutic approaches exists for patients with HO.

## Clinical messages

• HO can lead to joint range limitations, disrupting activities of daily living engagement and subsequent decline in quality of life.
• Approaches to address HO needs to be customized to the patient and to utilize an interdisciplinary team with an emphasis on physical and occupational therapy expertise to ameliorate disability.

## Introduction

Heterotopic ossification (HO) is defined as the presence of mature lamellar bone in damaged soft tissues where it does not typically exist (1–3). HO can occur at major joints, such as the elbow or hip, and it is most likely to occur after a severe burn or following a spinal cord injury, traumatic brain injury, fractures or traumatic amputations (1, 4). There is a rapidly developing area of study exploring HO prevalence in severe SARS-CoV-2 infection (5, 6). When HO develops, it not only adversely impacts success in activities of daily living and functional mobility but can also lead to a decline in overall life satisfaction and quality of life (1). The disruption due to HO can have a long-lasting impact on outcomes of patients across the spectrum of care, including an increase in hospitalizations, poor discharge outcomes, and prolonged rehabilitation follow-up care.

The etiology of HO is unknown (1, 2, 7–9). There may be a genetic predisposition and prior research has investigated the potential link between HO and fibrodysplasia ossificans progressive, a rare disorder in which progressive HO develops (4, 7). The known risk factors for the development of HO include prolonged immobilization with consistent pressure on the areas likely to develop HO, mobilization after prolonged immobilization, severity of injury or surface area after burn, sustained mechanical ventilation, severe inflammation, hypercalcemia, and local spasticity (9, 10). Chronic trauma affecting an area during dressing changes and stretching exercises while under anesthesia has been shown to be an additional risk factor (7). Patients who are intubated are also unable to communicate with healthcare professionals, which may lead to unrecognized and excessive pressure being placed on the joints at risk could lead to the development of HO (9, 11). Risk factors that increase HO secondary to a spinal cord injury exist if the injury is severe at either the cervical or thoracic level. Following elbow fractures, the development of HO is more likely after proximal radius fractures, extensive triad injuries, and fractures/dislocations than distal humerus fractures (12). While HO can develop after all types of burns, it is most likely when the burn covers more than 30% total body surface area, is a full thickness burn, and when it crosses any joint, including the elbow (10).

The initial clinical signs of HO are typically swelling, redness, and warmth at the joint, as well as pain and limited range of motion (13). These signs may develop as early as two months after trauma or surgery, but they can also appear after several years. A sign specific to the elbow is known as the “locking sign,” that is similar to a bony end feel and can aid clinicians in differentiating HO from elbow scar and contractures (9, 13). As HO progresses, it further restricts range of motion due to the increased development of ectopic bone (14). HO at the elbow is also associated with muscle weakness, ulnar nerve compression, and pressure ulcers (9).

Uncertainty exists about the use of therapy interventions following the development of HO, specifically with the lack of defined management (7, 9, 10, 14). For example, in 1986, Crawford and colleagues reviewed and analyzed the records of twelve patients diagnosed with heterotopic ossification at the elbow following a burn injury (15). They concluded that passive stretching, and passive range of motion exercises should be contraindicated following a diagnosis of heterotopic ossification, and active range of motion (AROM) should be done within the pain-free range. However, the authors failed to adequately explain their rationale. In another study, continuous passive range of motion (CPM) was used to increase range of motion in a patient with HO at the knees following a TBI (16). The patient, who also received indomethacin and aggressive pain management, regained significant ROM (60 degrees) in each knee.

Without evidence-supported therapeutic recommendations, rehabilitation clinicians are facing contradictory orders from physicians, and often feel helpless when deciding a course of treatment. There can be no treatment fidelity and predictive outcomes from confusing and contradictory orders. In an ideal world, clinicians would have confidence in their approach. However, the literature does not present a unified front to realize this ideal world.

Controversy surrounds the role of therapeutic interventions, unable todetermine which is better, active or passive range of motion to improve joint range in the presence of HO. Paradoxically, after surgical mitigation following the development of HO, experts often recommend any or all of the following approaches—continuous passive motion, active and passive range of motion performed by a therapist and static progressive splinting (17–19). In addition, authors of a case series describing the development of HO following significant total body surface area burns recommend gentle passive range while the patient is intubated and pain-free AROM when the patient is able to participate in therapy (1). Given the scant evidence in the literature and confusion of different mitigating interventions for HO a scoping review is the most appropriate method to provide an overview of all the therapeutic approaches that could address HO.

## Methods

Our aim of this scoping review is to clarify and summarize therapeutic approaches to HO to the elbow, with our research question: Are both active and passive range of motion exercises effective in maintaining or improving range of motion in people with heterotopic ossification? Utilizing the Arksey and O'Malley (20) methods, our scoping review and the Preferred Reporting Items for Systematic Reviews and Meta-Analyses (PRISMA) Extension for Scoping Reviews (20).

No protocol was found that met our research questions, we utilized a novel search. For the search strategy the following electronic databases were searched: the Cochrane Database of Systematic Reviews, PubMed, CINAHL, PsycINFO, Web of Science, and OTSeeker. To ensure the search was comprehensive, the authors also conducted searches of the following journals: Burns and Trauma, Burns Journal, Burns Open, and the Journal of Hand Therapy. The key search terms were “heterotopic ossification” combined with “spinal cord,” “traumatic brain injury,” “stoke,” “amputation,” “genetic disorder,” or “fracture” and “exercise,” “motion,” and “elbow.”

**Figure 1:**
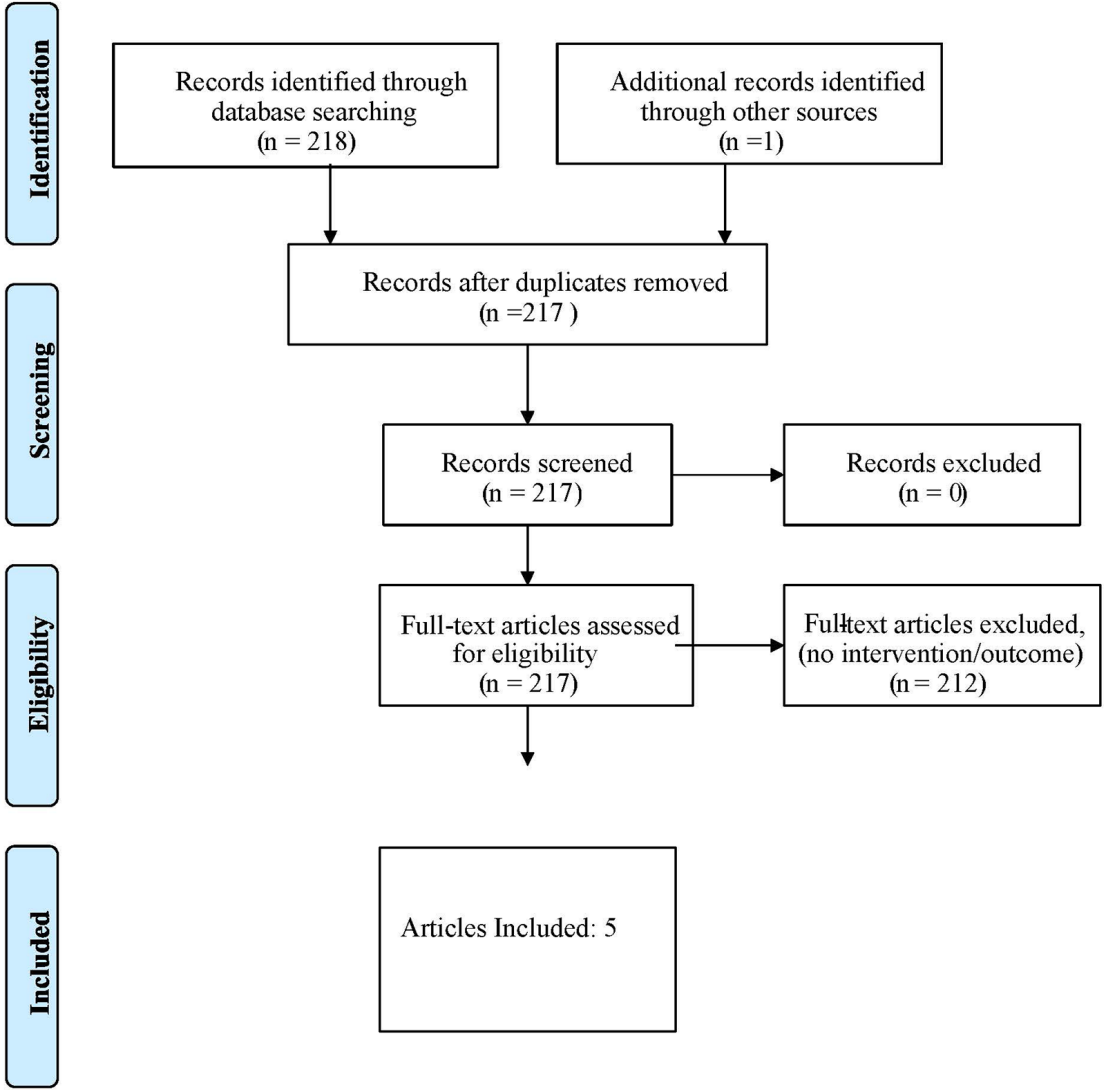
Flow diagram of review process according to the PRISMA extension for scoping reviews.

Due to the limited amount of research in this area, no restriction on date or publication type was applied. A summary of article exclusions with reasoning is presented in the Flow diagram of review process according to the PRISMA Extension for Scoping Reviews in Figure 1 (20). The search was initially conducted in 2018, but then reconducted to identify more recent publications in 2022. The selection process of articles was conducted by two members of the team, upon agreement a third member reviewed and came to an agreement of included set of articles. Originally 218 articles were found, one was removed due to duplication, 212 were removed as they did not meet the inclusion criteria containing no intervention or outcomes, leaving five remaining in the search. The team then assembled Table 1, which includes a summary for each article included in the review. This table includes details about the study design, intervention used, outcome measures and their respective results.

**Table 1:** Published studies included.

Study	Study design & diagnosis	Intervention	Outcome measures	Results
Coons et al. (21)	Level V Case Series Burn-associated HO *N* = 2 Participant 1: 37-year-old female (TBSA 83%) Participant 2: 23-year-old male (TBSA 72.5%)	Intensive passive range of motion—leaning on forearms for one to five minutes, 4–5 times per day or within pain tolerance for four months.	Active range of motion of joint affected by HO in degrees	Participant 1: 6 months post- +65⁰ at the right elbow +55⁰ at the left elbow at discharge 4-year post- Full bilateral ROM was at full flexion and lacking 10 degrees of full extension (10⁰ with no surgical release). Participant 2: +65⁰ on the left +25⁰ on right at 6 months post discharge. At 3-year follow up, bilateral ROM was at full extension and lacking 10 degrees of full extension (10⁰ with a surgical release)
Richards et al. (1)	Level V Case Series Burn-associated HO *N* = 3 Participant 1: 55-year-old male (TBSA 75%) Participant 2: 21-year-old male (TBSA 40%) Participant 3: 30-year-old male (TBSA 80%)	Active range of motion within the pain free range for each participant	Active range of motion of joint affected by HO in degrees	Participant 1: - right elbow: no return, ROM was 0–125° after required surgical release, - left elbow: surgical release scheduled Participant 2: +35° flexion at right elbow—surgical release scheduled 15–90° of active motion at left elbow—surgical release scheduled Participant 3: +35° active flexion in his right elbow and +20° in his left elbow
Crawford et al. (15)	Level V—Retrospective case series Burn—associated HO *N* = 12 Mean age = 35 (21–53) Mean TBSA is 49% (20%–85%)	Active range of motion within the pain free range for each participant	Active range of motion of joint affected by HO in degrees	3 participants recovered to functional range of motion. 3 participants recovered to normal range of motion. 6 participants required surgical excision. **No mention of specific gains in ROM
Peterson et al. (22)	Level V—Retrospective Case Series Burn-associated HO *N* = 18 (17 at the elbow) Mean age = 37 (21–61) Mean TBSA = 43% (8%–85%)	Active range of motion within the pain free range for each participant	Active range of motion of joint affected by HO in degrees	4 participants regained functional range of motion 6 participants regained normal range of motion These ten participants started with an average of 46 degrees of range of motion and ended with 124 degrees of range of motion after therapy. 8 participants had to have surgical excision due to unresponsiveness to therapy
Garland et al. (23)	Level IV—Retrospective Chart Review Trauma-associated HO 13 elbows joints in 9 patients	Passive range of motion under anesthesia	Active range of motion of joint affected by HO in degrees	Seven elbows were manipulated once, and six were manipulated twice. 6 of the patients (62%) gained an average of 47 degrees. Three of the patients did not gain or lost range of motion. Note: This study also examined HO in the hip. They also concluded that manipulation under anesthesia did not hasten progression of HO.

TBSA, total body surface area.

### Inclusion and exclusion criteria

Studies included were originally published in English, where the elbow was the joint of focus and a clearly defined therapeutic intervention was performed prior to or without definitive surgical excision of HO. Only studies that included pre-intervention and post-intervention measurements, either in terms of degrees gained, or in terms of functional level achieved, were included to ensure that changes were due to the intervention provided. Exclusion criteria included studies that did not include a therapeutic intervention, did not present outcome data relating to range of motion gained or lost as a result of performing active or passive range of motion, or only reported gains and/or losses in range of motion after surgical excision was performed.

## Results

The initial citation and abstract search yielded 219 articles related to HO. Duplicate articles and articles that did not meet the inclusion criteria were removed. The full-text versions of potential articles were retrieved, and the review team made further exclusions based on the predetermined inclusion and exclusion criteria. Five level V articles, as defined by the Oxford Centre for Evidence-Based Medicine, remained for analysis following review. Each article is summarized in an evidence table (Table 1) that displays the methods and major findings of therapy practices for rehabilitating a person with HO at the elbow.

### Passive range of motion

Two articles discussed passive range of motion as an intervention for HO at the elbow. One article reviewed 16 patients with a traumatic brain injury, nine of whom were diagnosed with HO (23). From those nine patients, a total of 13 elbows acquired heterotopic ossification. Eight of the 13 elbows (62%) gained an average of 47 degrees following manipulation under anesthesia. Five of the nine patients (55.5%) gained enough range of motion at the elbow to be considered functional in either one or both elbows, while four patients did not and required surgical intervention.

In 2012, Coons & Godleski reported on two patients who were diagnosed with HO at the elbow secondary to a burn injury. Both patients underwent an aggressive passive range of motion therapy protocol. One patient went from having less than 90 degrees of elbow flexion bilaterally to having full bilateral elbow flexion after 6 months of treatment, which resulted in the patient not requiring surgery to regain elbow range of motion. The second patient went from having 10 degrees of range of motion bilaterally (from 90 degrees to 100 degrees) to having an additional 10 degrees of extension at both elbows and 160 degrees of left elbow flexion and 110 degrees of right elbow flexion (left elbow = 80–160 degrees; right elbow = 80–110 degrees). The total range of motion improvement for this patient was 70 degrees at the left elbow and 20 degrees at the right elbow. This patient did have to undergo surgical excision and after three years, their elbow range of motion was functional bilaterally and they only lacked 10 degrees of terminal extension on both sides.

### Active range of motion

Three articles described using active range of motion within the client's pain free range to treat heterotopic ossification at the elbow. The Richards and Klaasen (1) case series reviews three patients with a diagnosis of heterotopic ossification at the elbow secondary to a burn injury. The patients who had extensive burns along with significant secondary complications, initially appear to have received passive range of motion, which was changed to active range of motion after diagnosis of HO. The patients went on to develop complete ankyloses that required surgical excision to regain range of motion.

The second article, Crawford et al. (15), examined 12 patients with HO at the elbow secondary to burn injury. Of the 12 patients, six (50%) responded to the active range of motion treatment with spontaneous resolution of HO at the elbow, three of which had functional range of motion and the other three gained full range of motion. The other six patients who did not respond to the active range of motion protocol underwent surgical excision at the elbow to regain range of motion.

The third article (22), reported the utilization of active range of motion within the pain free range to treat heterotopic ossification at the elbow following a burn injury. The authors found 10 of the 18 patients (55.5%) had an approximate 78 degree increase in range of motion after 6 months. Six of those 10 returned to normal range of motion and the other four gained functional range of motion. The remaining eight patients did not respond to the active range of motion protocol and required surgical excision to regain range of motion.

### PEDro scale

The Physiotherapy evidence database PEDro scale was used to measure the validity of the methodological quality of each article being reviewed in the current study (25). According to the PEDro scale, if a study receives 9–10 points it is considered to have an excellent level of evidence, 6–8 points is considered good, 4–5 points is considered fair, and a study that has below 4 points it is considered to have a poor level of evidence. All authors of this review were in complete agreement as to the PEDro score assigned to each of the five studies. Each author concluded that all five articles received a PEDro score of three, which corresponds to poor level of evidence.

## Discussion

Limited evidence was found to support the use of either active or passive range of motion to rehabilitate the elbow once a diagnosis of HO. Two of the five studies included reported the use of passive range of motion increased overall range of motion resulting in 45.5% of the total number of patients being able to forego surgery. In contrast, the other three studies reported on patients who received therapy protocols of active range of motion only at the elbow after the diagnosis of HO. In these three studies, 50% of the patients reviewed were able to forego surgery while the other half underwent surgery to regain range of motion. These results are statistically neutral and clearly demonstrate am inclusiveness in either approach.

The authors of this review found numerous articles that referenced Crawford et al. (15) as the basis for recommending active range of motion as the only type of movement done at the elbow once a person is diagnosed with heterotopic ossification, yet 50% of the patients reviewed in the Crawford paper failed to respond to this conservative treatment and required surgery. Further challenging the basis of this approach, is a subsequent case series completed at the same hospital by Peterson et al. (22), which may have included some of the patients initially reported by Crawford, et al. (15). Several of the patients described were very similar in both studies leading to samples that may not have been entirely independent of each other.

In another literature review published in 2006, the authors concluded that there was no scientific evidence that controlled (passive) range of motion or splinting caused heterotopic ossification (HO) (3). In addition, based on their review, the authors advocated for both active and passive exercises along with static progressive splinting to mitigate the development of and as interventions for HO (3). However, a more recent review concludes that it is unclear if passive range of motion progresses development of HO or improves range-of-motion after development. The authors also conclude that direct comparison trials are lacking and recommendations for or against passive range of motion are often based on clinician preferences and not evidence (3).

None of the articles included in this scoping review make attempt to define passive range of motion or differentiate passive range of motion from passive stretching. In addition, authors of a review of HO following spinal cord injury recommend early joint mobilization as primary prevention of HO (24). Another review recommends passive range of motion and suggests that aggressive joint manipulation as early treatment of HO could lead to further development of HO (24). A clear picture of the appropriate approach was not given.

### Limitations

A clear limitation of this study is the few articles examining the therapeutic approach to address HO in the elbow, yet this is indicative of the amount of research that has been completed and published in peer-reviewed journals on this topic. Well-designed research needs to be conducted. Due to the circumstances in which HO is formed, future studies need to establish a well-conceived recruitment strategy. This review focused on HO in the elbow solely, and that may have limited the number of articles that met the inclusion criteria. An additional study limitation is the absence of clear definitions of passive range of motion vs. passive stretching. Each study that does not provide a clear definition of this concept is hampering the clinical picture.

### Clinical implications

The clinical implications of this scoping review are that therapeutic approaches to address HO require a team discussion and approach, including physicians, occupational therapists, and physical therapists. The clinical team should evaluate, discuss and decide the best course action to address the limitations caused be the presence of HO.

### Research implications

The research implications of this scoping review have provided some useful information for future research in this area. This review has revealed that only very few studies have examined therapeutic approaches to HO. These results should urge researchers to pursue studying pragmatic HO approaches in order to provide clinical guidance. Additionally, clearly defining the approach used is crucial for dissemination and implementation of research. The lack of clarity in the articles reviewed did not alleviate confusion, but added to it. How can physical and occupational therapists possibly be expected to make an evidence-based clinical decision, if none exists? An examination of what therapists are doing in the clinic to address HO is required. Followed by utilizing experience-based practice of experts to develop a testable protocol and establish a clinical practice rooted in evidence. Next, developing a well-designed randomized controlled trial can finally establish the gold standard to approach HO in the elbow.

## Conclusion

Heterotopic ossification affects patients with central nervous system disorders, orthopedic or neurological injuries, severe respiratory diseases, and burns. HO impedes function and independence due to joint limitations from the ossification of soft tissue. Occupational and physical therapy practitioners need guidance in how to manage joints affected by HO. A surgical approach is commonly used to return function, however this option may not be possible for everyone, a gold standard for a conservative approach is needed. This scoping review attempted to summarize and identify current supported evidence on the appropriate approach. While each person diagnosed with heterotopic ossification will react differently to therapy, physicians, occupational therapists, and physical therapists should evaluate, discuss, and decide whether a therapy protocol of active range of motion, passive range of motion, or a combination of the two should be used. Basing clinical decisions only on evidence provided by case studies and retrospective reviews is limiting. Future research is necessary to determine the clinical value of passive vs. active range of motion for patient's diagnosis with heterotopic ossification at the elbow.
